# Evaluation and reconditioning of donor organs for transplantation through *ex vivo* lung perfusion

**DOI:** 10.31744/einstein_journal/2019AO4288

**Published:** 2019-07-12

**Authors:** Luis Gustavo Abdalla, Karina Andrighetti de Oliveira-Braga, Lucas Matos Fernandes, Marcos Naoyuki Samano, Paula Refinetti Camerini, Paulo Manuel Pêgo-Fernandes

**Affiliations:** 1 Hospital Israelita Albert Einstein Hospital Israelita Albert Einstein São PauloSP Brazil Hospital Israelita Albert Einstein, São Paulo, SP, Brazil.; 2 Universidade de São Paulo Universidade de São Paulo Faculdade de Medicina São PauloSP Brazil Faculdade de Medicina, Universidade de São Paulo, São Paulo, SP, Brazil.

**Keywords:** Lung transplantation, Organ preservation, Brain death, Transplante de pulmão, Preservação de órgãos, Morte encefálica

## Abstract

**Objective:**

To assess the feasibility and impact of *ex vivo* lung perfusion with hyperoncotic solution (Steen Solution^™^) in the utilization of these organs in Brazil.

**Methods:**

In this prospective study, we subjected five lungs considered to be high risk for transplantation to 4 hours of *ex vivo* lung perfusion, with evaluation of oxygenation capacity. High-risk donor lungs were defined by specific criteria, including inflammatory infiltrates, pulmonary edema and partial pressure of arterial oxygen less than 300mmHg (inspired oxygen fraction of 100%).

**Results:**

During reperfusion, the mean partial pressure of arterial oxygen (inspired oxygen fraction of 100%) of the lungs did not change significantly (p=0.315). In the first hour, the mean partial pressure of arterial oxygen was 302.7mmHg (±127.66mmHg); in the second hour, 214.2mmHg (±94.12mmHg); in the third hour, 214.4mmHg (±99.70mmHg); and in the fourth hour, 217.7mmHg (±73.93mmHg). Plasma levels of lactate and glucose remained stable during perfusion, with no statistical difference between the moments studied (p=0.216).

**Conclusion:**

*Ex vivo* lung perfusion was reproduced in our center and ensured the preservation of lungs during the study period, which was 4 hours. The technique did not provide enough improvement for indicating organs for transplantation; therefore, it did not impact on use of these organs.

## RESUMO

**Objetivo:**

Avaliar a exequibilidade e o impacto da perfusão pulmonar *ex vivo* com solução hiperoncótica (Steen Solution^™^) na taxa de utilização desses órgãos no Brasil.

**Métodos:**

Neste estudo prospectivo, submetemos cinco pulmões considerados de alto risco para o transplante a 4 horas de perfusão pulmonar *ex vivo*, com avaliação da capacidade de oxigenação pulmonar. Os pulmões de doadores de alto risco foram definidos por critérios específicos, incluindo infiltrado inflamatório, edema pulmonar e pressão parcial de oxigênio arterial inferior a 300mmHg (fração inspirada de oxigênio de 100%).

**Resultados:**

Durante a reperfusão, a pressão parcial de oxigênio arterial (fração inspirada de oxigênio de 100%) média dos pulmões não sofreu alteração significativa (p=0,315). Na primeira hora, a pressão parcial de oxigênio arterial média foi de 302,7mmHg (±127,66mmHg); na segunda, 214,2mmHg (±94,12mmHg); na terceira, 214,4mmHg (±99,70mmHg); e na quarta, 217,7mmHg (±73,93mmHg). Os níveis plasmáticos de lactato e glicose se mantiveram estáveis ao longo da perfusão, sem diferença estatística na comparação entre os momentos estudados (p=0,216).

**Conclusão:**

A perfusão pulmonar *ex vivo* foi reproduzida em nosso centro e garantiu a preservação de pulmões durante o período de estudo, que foi de 4 horas. A técnica não promoveu melhora suficiente para indicação do órgão para o transplante e, portanto, não impactou na taxa de utilização desses órgãos.

## INTRODUCTION

Currently, transplantation is the main therapeutic modality for patients with end-stage pulmonary disease. One of the limiting factors for performing lung transplant is the offer of viable organs. This is because the lungs are extremely susceptible to the deleterious effects of brain death and of complications from intensive care units (ICU) stay (prolonged mechanical ventilation, nosocomial pneumonia, barotrauma, and excessive infusion of crystalloids), making them unviable for a transplant. Presently, worldwide data show that only 15 to 20% of lungs offered are utilized.^(^ ^1^ ^–^ ^3^ ^)^

Over the last years, a series of studies related to *ex vivo* pulmonary perfusion (EVLP) with a highly oncotic solution (Steen Solution^™^, Xvivo Perfusion, Gothenburg, Sweden) has impacted lung transplant programs positively, with the perspective of increasing the pool of donors and decreasing the mortality of patients on the waiting list. *Ex vivo* pulmonary perfusion can be used to recondition high-risk lungs, and is characterized, among other criteria, by the presence of edema and a ratio of partial oxygen pressure/ inspired oxygen fraction (PO_2_/FiO_2_) 100% <300mmHg.^(^ ^4^ ^–^ ^8^ ^)^ Furthermore, by means of this technique, it is possible to evaluate and use lungs from donors after circulatory arrest.^(^ ^3^ ^,^ ^9^ ^–^ ^11^ ^)^ In EVLP, the lungs are isolated and submitted to perfusion with a highly oncotic solution, which reduces tissue edema and contributes to the clearance of cells and inflammatory mediators, improving the organ's general condition. Recent studies have shown postoperative results similar to those who received post-EVLP lungs in comparison with those who received lungs considered ideal.^(^ ^6^ ^,^ ^12^ ^)^

*Ex vivo* pulmonary perfusion was initially employed in Sweden, utilizing donors after circulatory death (DCD), with encouraging results and survival comparable to conventional lung transplants.^(^ ^13^ ^)^ Cypel et al.,^(^ ^14^ ^)^ applied this technique with modifications for the reconditioning of lungs considered to be high risk from brain dead donnors (BDD) and DCD, maintaining the organs stable during the EVLP, and with post-transplant results comparable to those of transplantations using selected organ donors based on conventional criteria. The incidence of primary dysfunction of the graft in this cohort was only 15% in comparison with 30% in the group of conventional lung transplants. *Ex vivo* pulmonary perfusion has been implemented in clinical practice by several transplantation centers, especially in North America and Europe. Some centers increased the number of transplants from 20 to 40%.^(^ ^15^ ^)^

Nevertheless, this is a technique with highly complex logistics, which depends on the clinical reality and procurement methods of each country. A few studies were published with the objective of clarifying the main stages and challenges for implementing EVLP programs. In Brazil, clinical and experimental studies utilized the EVLP technique with the objective of both evaluation and pulmonary reconditioning.^(^ ^4^ ^,^ ^16^ ^–^ ^18^ ^)^

## OBJECTIVE

To evaluate the viability of *ex vivo* pulmonary perfusion in high-risk lungs donated for transplantation and determine if the *ex vivo* pulmonary perfusion increases the utilization of these organs.

## METHODS

This is a cross-sectional prospective study, carried out at a single center after approval by the *Conselho Nacional de Saúde – Comissão Nacional de Ética em Pesquisa* (CONEP) [National Health Council - National Research Ethics Committee] (CONEP registry, process with approval from the Research Ethics Committee, opinion 965.945, CAAE: 41729615.2.0000.0071).

The sample was composed of human donors with brain death rejected for conventional transplant due to the presence of PaO_2_/FiO_2_ <300mmHg, or lungs presenting with infiltrates upon radiological evaluation.

The inclusion criteria used were age <50 years; smoking <20 packs-years; absence of thoracic trauma; absence of signs of aspiration or pus on bronchoscopy; arterial gases with PaO_2_ <300mmHg (FiO_2_ 100% and positive end-expiratory pressure − PEEP 5cmH_2_O).

Between January 2016 and December 2016, five harvests were made for the pulmonary reconditioning protocol. The lungs were obtained and perfused with a lung preservation solution, Perfadex^®^ (Vitrolife, Gothenburg, Sweden), stored in a specific bag at 4°C, and sent to the Operating Room of *Hospital Israelita Albert Einstein*, in the city of Sao Paulo (State of Sao Paulo).

**Figure 1:**
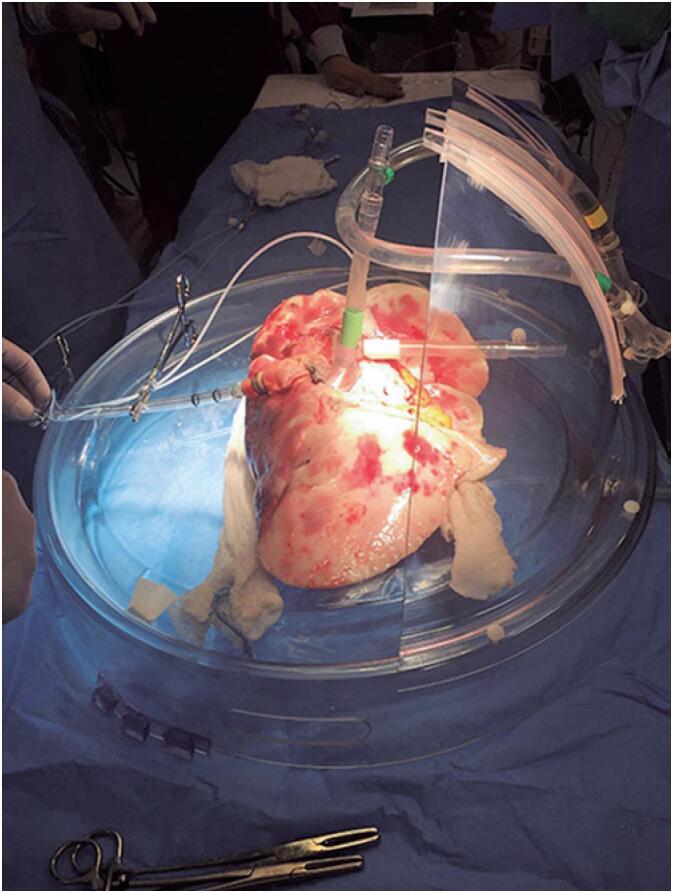
Lung of human donor placed in an *ex vivo* chamber

The pulmonary artery and pulmonary veins were connected to specific cannulae, and the lung block was kept in a perfusion chamber (Xvivo Perfusion, Gothenburg, Sweden) (Figure 1).

**Figure 2:**
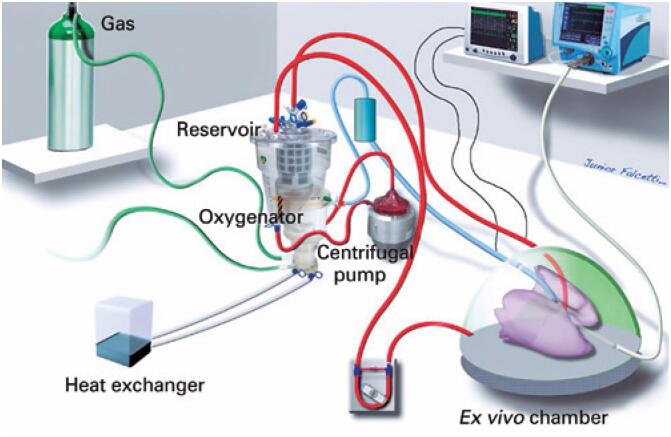
Scheme of *ex vivo* pulmonary perfusion system

The perfusion system consisted of a membrane oxygenator, heat exchanger, leukocyte filter, and centrifugal pump (Maquet, Toronto, Canada), as shown in figure 2.

The perfusate leaves the lungs by means of cannulation of the left atrium and enters the reservoir. A centrifugal pump drives the perfusate to the oxygenator and heat exchanger, where deoxygenation occurs and it is warmed up to normothermia. Then the perfusate passes through a leukocyte filter before returning to the lungs through the cannulation made in the pulmonary artery.

The system was filled with 1.5L of perfusion solution, Steen Solution^™^ (Vitrolife), and 500mg of methylprednisolone, 500/500mg of cilastatin sodium + imipenem, and heparin (3,000IU). The flow established was 40% of cardiac output, with ventilation within physiological standards: tidal volume (TV) of 6mL/kg, respiratory rate 7 cycles/minute, positive end-expiratory pressure of 5cmH_2_O, and FiO_2_ of 21%. The increase in flow, rewarming, and ventilation followed the protocol described by Cypel et al.^(^ ^14^ ^)^

The programmed time of perfusion was 4 hours, with evaluation of ventilatory and blood gas parameters at the end of each hour. At the end of the third hour, the perfusion was interrupted in the organs in which there was no perspective of improving lung function or ventilatory mechanics with the purpose of transplantation of the organ. At the time of evaluation, the ventilator was adjusted for a TV of 10mL/kg weight, respiratory rate of 10 cycles/minute, positive end-expiratory pressure of 5cmH_2_O, and FiO_2_ of 100%. A sample of the perfusate was collected from the outlet of the pulmonary vein and pulmonary artery to test blood gases, including the values of partial pressure of carbon dioxide (PaCO_2_), mixed venous pressure of carbon dioxide, PaO_2_, mixed venous pressure of oxygen, and lactate and glucose levels. The ventilatory parameters collected were peak pressure, plateau pressure, mean airway pressure, static complacency, and TV. Also monitored were the parameters of mean pulmonary artery pressure and perfusion flow.

The data were presented as means accompanied by the respective standard deviations. Normal distribution and homogeneity of the variances were assessed, respectively, with the Shapiro-Wilk and Levene tests. To compare means, repeated measures of analysis of variance (ANOVA) and the paired Student's *t* test were used. When it was necessary to perform multiple comparisons of means, the Bonferroni test was used. A probability of error type I (α) of 0.05 was considered in all inferential analyses. The descriptive and inferential statistical analyses were performed using the software Statistical Package for the Social Sciences (SPSS) for Windows, version 21.

## RESULTS

This study included five pulmonary blocks from donors with a mean age of 40 years; two came from men, and three from women. Of the five lungs obtained, three were rejected for conventional transplantation due to unsatisfactory arterial gases (PaO_2_ lower than 300mmHg with FiO_2_ of 100%, and PEEP of 5cmH_2_O). Two lungs, although presenting with satisfactory blood gases, were rejected due to the presence of pneumonia noticed in the tissue sample in one case, and persistent infiltrate after EVLP in the other. The primary causes of death were hemorrhagic stroke (three donors) and subarachnoid hemorrhage (two donors). Table 1 displays the demographic and clinical data of the donors.

**Table 1:** Characteristics of selected donors

Age	Sex	Cause of death	Days on MV	Final PaO_2_ (mmHg)
43	Male	Head trauma	2	182
44	Female	Subarachnoid hemorrhage	5	428
27	Male	Head trauma	5	502
46	Female	Subarachnoid hemorrhage	4	205
50	Male	Head trauma	4	330

MV: mechanical ventilation; PaO_2_: partial pressure of oxygen.

**Figure 3:**
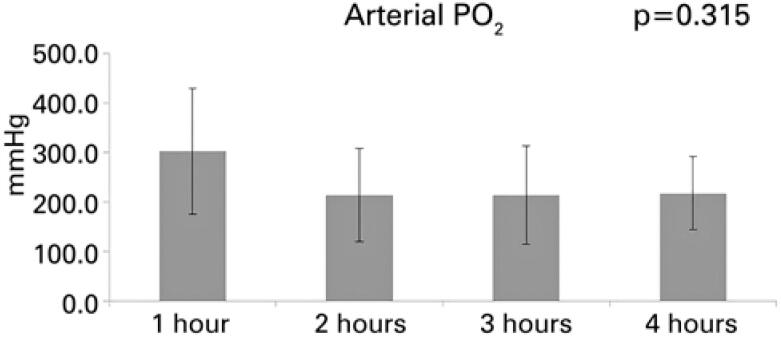
Pressure of arterial oxygen during *ex vivo* pulmonary perfusion

During reperfusion, the mean arterial PaO_2_ (FiO_2_ 100%) of lungs did not undergo any significant alteration (p=0.315). During the first hour, the mean arterial PaO_2_ was 302.7mmHg (±127.66mmHg); in the second hour, 214.2mmHg (±94.12mmHg); in the third hour, 214.4mmHg (±99.70mmHg); and in the fourth hour, 217.7mmHg (±73.93mmHg), as shown in figure 3.

Considering the data isolated from each perfusion, in four cases the lungs evolved with a decrease in PaO_2_ throughout the period, and in one case, there was an increase in PaO_2_ (Table 2).

**Table 2:** Isolated progression of partial pressure of arterial oxygen (PaO_2_) throughout perfusion

Arterial PaO_2_ (mmHg)				
Case	First hour	Second hour	Third hour	Fourth hour
1	316.0	308.0	217.0	148.5
2	388.3	316.0	375.8	231.0
3	454.0	142.0	148.0	327.0
4	136.0	*	213.0	233.0
5	219.0	111.0	118.0	*

* Data not collected due to problems related to verification of the equipment used.

The oxygenation capacity, taking into consideration the arterial PaO_2_ and the venous PaO_2_ (ΔPO_2_), showed no statistical difference between the times studied (p=0.116). A mean was noted of 196mmHg (±137.31mmHg), 78mmHg (±150.69mmHg), 78.8mmHg (±130.69mmHg), and 217.7mmHg (±97.19mmHg), respectively, during the 4 hours of evaluation.

Plasma levels of lactate and glucose were stable throughout the perfusion, with no statistical difference between the times studied (p=0.216).

## DISCUSSION

The utilization of perfusion to multiple organs was originally proposed in the 1930's by Carrel et al.,^(^ ^19^ ^)^ and specifically for lungs, in the 1970's, by Jirsch et al.^(^ ^20^ ^)^ In both cases, the objective was to prolong the preservation of the organ in cases of procurement over long distances. Nonetheless, at that time, the technological difficulties hindered the maintenance of integrity of the alveolar capillary barrier, leading to formation of edema and increased pulmonary vascular resistance during the procedure. Over the last years, in an attempt to evaluate organs from donors with cardiac arrest, Steen et al.,^(^ ^13^ ^,^ ^21^ ^)^ developed a perfusion solution (Steen Solution^™^), with the intent of optimizing the internal colloid osmotic pressure, besides furnishing the necessary nutrients for perfusion over long periods.

The objective of EVLP is to provide a safe platform for detailed evaluation of lungs donated for transplantation, maintaining the organ in specific physiologic conditions, and for reconditioning organs initially considered inadequate for transplantation. In this way, the EVLP may be applied in two distinct contexts: to preserve and evaluate the DCD organ, or to recondition organs considered borderline BDD or DCD.

In the present study, five lungs were submitted to EVLP with Steen Solution^™^. The lungs were maintained in *ex vivo* perfusion for 4 hours, and the general physiological condition of the organ was preserved. Despite the results being positive in preservation and evaluation of organs, the lungs used in this protocol came from BDD patients and were considered high-risk - and therefore, required functional improvement and edema reduction.

The evaluation and organ preservation studies, without necessarily promoting reconditioning, are especially important when DCD are used. The first protocols developed by Steen et al.,^(^ ^13^ ^)^ evaluated the pulmonary function of organs from DCD patients during short periods of perfusion (60 minutes), followed by transplantation. Since the first transplant with DCD after the use of EVLP, in 2007, the number of transplants with DCD grew exponentially, and in all cases, EVLP was used.^(^ ^3^ ^,^ ^13^ ^,^ ^22^ ^,^ ^23^ ^)^ The lungs from DCD, in general, are better preserved due to the absence of deleterious lung effects of brain death relative to the lungs of BDD, rejected for conventional transplantation and elected for EVLP. Some studies pointed to positive results in transplants with DCD, even without the previous use of EVLP.^(^ ^24^ ^)^

The use of EVLP in protocols of reconditioning for a prolonged period was described by Cypel et al.,^(^ ^25^ ^)^ in 2008. Our protocol utilized the EVLP with the modifications in the technique suggested by the Toronto group. We employed a ventilation strategy and protective perfusion, acellular perfusate (without the addition of packed red blood cells) and left atrium pressure of 3 to 5mmHg. Such modifications allowed a prolonged perfusion, defined herein in 4 hours, needed for organ reconditioning. However, the captured organs had a high level of compromise in general, due to prolonged time in brain death they had been exposed to within the current dynamic of the Brazilian National Health System (SUS - *Sistema Único de Saúde*), where it is known that the diagnosis of brain death is delayed, and the routine of confirmatory tests is slow.

In Brazil, the technique was applied clinically at the *Instituto do Coração* (Incor), in the city of São Paulo (State of São Paulo), with results similar to those of this study.^(^ ^4^ ^)^ The lungs evaluated remained in physiological conditions of preservation; but the protocol was not effective for promoting improvement in pulmonary function, making the transplant unviable. The authors attributed the result to the use of organs with lesions and pronounced physiological alterations, as well as to the restricted number (n=5) of perfusions conducted. Thus, we and the team from Incor, had difficulties in procurement of organs, since even when using extended and more permissive inclusion criteria for a donor in the study, the number of procurement was low, due to the complications that donors face in hospital setting during prolonged periods. The number of perfusions that we performed was also small and could be correlated with the findings. Taking into consideration the time limits for importation and validity of the materials acquired for EVLP, the remaining time left for inclusion of lungs into the study is small, which is aggravated by the scarcity of donors. Currently, most groups that clinically apply the technique increased the pool of donors using DCD. However, it is still unviable in Brazil due to the present laws, which only allow the use of BDD.

Currently, the use of EVLP for evaluation and reconditioning of organs is expanding. A series of clinical studies is underway in Europe and United States.^(^ ^12^ ^)^ Nonetheless, EVLP is effective for donors with less pronounced lesions than those frequently found in our donors.

## CONCLUSION

The *ex vivo* lung perfusion was reproduced in our center and guaranteed the preservation of the lungs during the study period, which was 4 hours. Nevertheless, it did not promote enough improvement for indication of the organ for transplant.
