# *Erratum* : Evaluation and reconditioning of donor organs for transplantation through *ex vivo* lung perfusion

**DOI:** 10.31744/einstein_journal/2019AO4288E

**Published:** 2019-12-03

**Authors:** Luis Gustavo Abdalla, Karina Andrighetti de Oliveira-Braga, Lucas Matos Fernandes, Marcos Naoyuki Samano, Paula Refinetti Camerini, Paulo Manuel Pêgo-Fernandes

**Affiliations:** 1 Hospital Israelita Albert Einstein Hospital Israelita Albert Einstein São PauloSP Brazil Hospital Israelita Albert Einstein, São Paulo, SP, Brazil.; 2 Universidade de São Paulo Universidade de São Paulo Faculdade de Medicina São PauloSP Brazil Faculdade de Medicina, Universidade de São Paulo, São Paulo, SP, Brazil.

The authors of the paper “Evaluation and reconditioning of donor organs for transplantation through ex vivo lung perfusion”, DOI number: 10.31744/einstein_journal/2019AO4288, published in einstein (São Paulo). 2019;17(4):eAO4288 report that the study received funding from the Brazilian Ministry of Health through the PROADIS-SUS Program (Process/SIPAR Nº 25000.014875/2015-12 part of the term Nº 01/2014, published in Diário Oficial da União on May 29, 2015). During the submission process to einstein (São Paulo) the authors failed to disclose the information about funding.

